# Investigation of Building Materials’ Radioactivity in a Historical Building—A Case Study

**DOI:** 10.3390/ma15196876

**Published:** 2022-10-03

**Authors:** Adriana Estokova, Eva Singovszka, Marian Vertal

**Affiliations:** 1 Institute of Sustainable and Circular Construction, Faculty of Civil Engineering, Technical University of Kosice, Vysokoskolská 4, 042 00 Kosice, Slovakia; 2 Department of Strategic Development, Municipality of Kosice, Trieda SNP 48/A, 040 11 Kosice, Slovakia; 3 Institute of Architectural Engineering, Faculty of Civil Engineering, Technical University of Kosice, Vysokoskolská 4, 042 00 Kosice, Slovakia

**Keywords:** gamma index, building materials, NORM, natural radionuclides, natural radiation, ^226^Ra, ^40^K, ^232^Th

## Abstract

The paper investigates a possible hazard originating from natural radionuclides in building materials in a selected historical building being reconstructed for housing. Both outdoor and indoor risks were evaluated through the radiological indices and estimated doses, based on measured activities of natural radionuclides in stone and brick materials of the building. The average measured activity concentrations of radionuclides were 7.32 Bq/kg for ^226^Ra, 40.05 Bq/kg for ^232^Th, and 546.64 Bq/kg for ^40^K radionuclides. The average total activity concentration in building materials (594.0 Bq/kg) exceeded the world average value. A correlation was found between the potassium content in the building material samples and the total activity of radionuclides. The gamma indices, *Iγ,* calculated for the samples, ranged in an interval of 0.26–0.60, not exceeding the restricted limit for bulk materials *Iγ* = 1. The average annual effective dose due to building materials was 0.53 mSv/y, which does not exceed the limit (1 mSv/y), however, it contributes to a gamma dose excess that is higher than recommended (0.3 mSv/y at the most). The bricks were responsible for a higher level of natural radiation than natural stone material. Nevertheless, based on the radiation protection requirements, it can be concluded that the building can be used for residential purposes after the reconstruction, as no significant human health impact is expected due to the radioactivity of building materials.

## 1. Introduction

The radiation to which the human population is exposed comes from many diverse sources. Some of these sources are natural; others are the result of human activities. The radiation from natural sources includes cosmic radiation, external radiation from radionuclides in Earth’s crust, and internal radiation from radionuclides inhaled or ingested and retained in the body [1,2]. The magnitude of these natural exposures depends on geographical location and on some human activities [1,2]. Height above sea level affects the dose rate from cosmic radiation; radiation from the ground depends on the local geology [3]. A significant part of the total dose contribution in the form of natural sources comes from terrestrial gamma radionuclides [2]. Nuclides with half-lives comparable with the age of the Earth or their corresponding decay products, existing in terrestrial materials, such as ^40^K, ^226^Ra, and ^232^Th radionuclides, are of great interest [1,2]. The human population worldwide receives an average annual radiation dose of 2.4 mSv/y, about 80% of which comes from naturally occurring radionuclides, while the remaining part is largely due to artificial sources of which fallout radionuclides account for only 0.4% [2].

Since most people spend about 80% of their time indoors, controlling the natural ionizing radiation in buildings is of great importance. One of the main sources of the indoor radiation is represented by building materials as building materials contain natural radionuclides [4]. Natural radioactivity of building materials originating from natural sources (soil, rock) is connected mainly with the radium (^226^Ra), thorium (^232^Th), and potassium (^40^K) radionuclides [5]. The built-in materials can cause both external and internal exposures indoors. The external exposure is caused by gamma radiation resulting from the decay of the radionuclides present in material; the internal exposure is caused by inhalation of the decay products of radionuclides present in materials, e.g., radon gas [6]. The level of radon concentrations, which can originate not only from building materials, but also from water and subsoil, is a non-negligible factor when evaluating indoor spaces. It is the subsoil that is often the most significant source of the presence of radon in the indoor environment [7,8]. Knowledge of hazards is required to take protective precautions to decrease the exposure of the population to ionizing radiation [9].

The main effects of ionizing radiation on living organisms are cell death, loss of reproductive capacity, or mutation. However, such effects depend on several factors, with the dose rate and the linear energy transfer (LET) of the radiation being the most important. The dose rate is the delivery of dose per unit time and the absorbed dose is typically measured in Grays, Gy, where 1 Gy = 1 J/kg [10]. The higher the dose rate, the greater the cell damage. Exposure to gamma radiation in the indoor environment would result in low doses of radiation. Recent advances in the knowledge of the mechanisms underlying the biological effects of low doses have shown that low radiation dose effects are mechanistically different to high radiation dose effects, with low radiation dose effects being similar to those of some chemicals in the environment. Thus, results under mixed exposures to radiation and chemicals may not be predictable for human health, by the consideration of single agent effects. It has been observed that the risk of increase in cancer incidence caused by low-dose radiation is low, but recent epidemiological studies have indicated elevated risks of non-cancer diseases (e.g., perturbation of immune function or induction of inflammatory reactions with disease) at low doses below 1–2 Gy, and in some cases much lower, although the mechanisms are still unclear and the estimation of risks remains problematic [11].

Natural radioactivity in building materials is measured as the activity concentration of the ^40^K, ^226^Ra, and ^232^Th radionuclides [12]. Since the distribution of ^226^Ra, ^232^Th, and ^40^K in building materials is not uniform, the real radioactivity levels in building materials can be assessed in the form of a single quantity by using several radiological indices [13]. The activity concentration index (gamma index) *Iγ*, including activity concentrations of all three radionuclides, was proposed by the European Commission and it is most often used to assess the dose level of external gamma radiation from building materials [14]. The activity concentration index shall not exceed the defined values (e.g., for bulk materials *Iγ* = 1) to ensure that the annual dose criterion of 1 mSv is met [14]. However, based on the radiation protection principles in the EU, controls are recommended for building materials contributing to overall dose by the value of 0.3–1 mSv/y [14] which corresponds to *Iγ* = 0.5–1 for bulk materials. Besides the activity concentration index, several others have been developed over the years to evaluate the radiation exposure due to building material, e.g., alpha index [10], indoor and outdoor hazard indices [15], different dose parameters [16,17,18]. Several studies have been presented concerning the natural radioactivity of building materials [19,20,21,22,23,24], based on which it can be concluded that conventional building materials do not pose a radiological hazard due to gamma radiation and rarely exceed the EU annual dose criterion for radiation protection. The worldwide average activity concentrations in building materials are 35 Bq/kg for ^226^Ra, 30 Bq/kg for ^232^Th, and 400 Bq/kg for ^40^K which correspond to a dose of 0.25 mSv/y [14]. However, a significantly higher level has been detected in some countries, such as Iran [25], Cameroon [26], or Chad [27].

On the other hand, the sustainability and circular economy principles require waste recycling or their re-use which leads to more significant incorporation of various industrial wastes into building materials. This is mainly connected with concrete and cement composites, thus representing more sustainable materials [28,29,30,31]. Waste often has a higher level of radioactivity than natural materials and therefore there is a need to monitor not only conventional but also these new sustainable materials in order to guarantee their safety when applied to the indoor environment [32].

Currently, several studies are devoted to the natural radioactivity of building materials as well as to the monitoring of the occurrence of radon in the interior, but there is rarely information on radiation exposure in historical buildings or the study of the radioactivity of historical materials. At present, in terms of cultural heritage, great emphasis is placed on the protection and restoration of historical site buildings that can be found not only in the historical centers of regional, district towns and surrounding villages but also in remote places [33,34]. The fact is that in the past, one was forced to build dwellings of the materials that were available and they were not always investigated for the potential radiological risk [35].

In this research, natural radioactivity of building materials from a selected UNESCO historical building in Slovakia was studied in order to assess the radiological impact of the historical building materials. Determination of the activity levels of the radionuclides and consequent evaluation of the building materials were performed in a historical building which was reconstructed for the purpose of its further use for housing. Radiation measurements of building materials aimed to determine the dose of radiation exposure from materials, and to evaluate the possible health risks posed by radiation.

## 2. Materials and Methods

**Figure 1:**
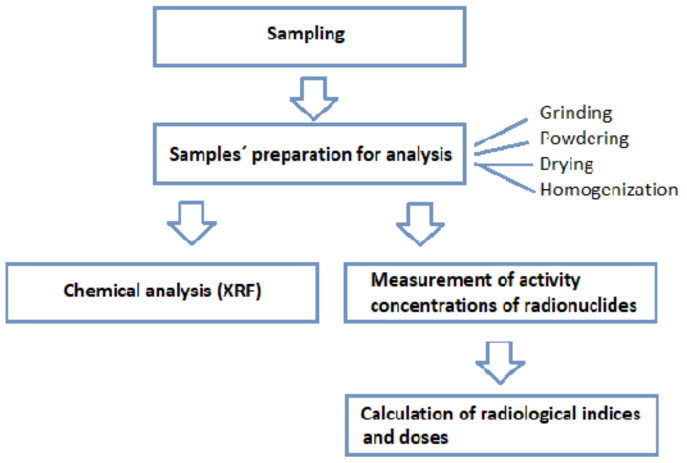
Schema of the steps and tools in the research.

The research of radioactivity of building materials in this study was carried out in the historic building in Master Paul’s Square of the UNESCO city Levoča, Slovakia. More detailed characteristics of the historical building are given in Section 2.1. Part of this building was undergoing reconstruction for residential purposes and building materials, represented by brick and stone samples, were collected during the reconstruction period as described in Section 2.2. After preparing the material samples (Section 2.3), chemical analysis was performed to determine the basic chemical composition of the brick and stone samples as well as to estimate the potassium radionuclide content based on the total potassium concentrations. The activity concentrations of radionuclides (^226^Ra, ^232^Th, and ^40^K) were measured in powdered samples of stone and brick materials using gamma spectrometry (Section 2.5). Measured values of the activity concentrations of radionuclides were used for the estimation of the external gamma exposure due to building materials by calculation and evaluation of various radiological indices and doses as described in more detail in Section 2.5. The general diagram of the research procedure is given in Figure 1.

### 2.1. Historical Building under Study

**Figure 2:**
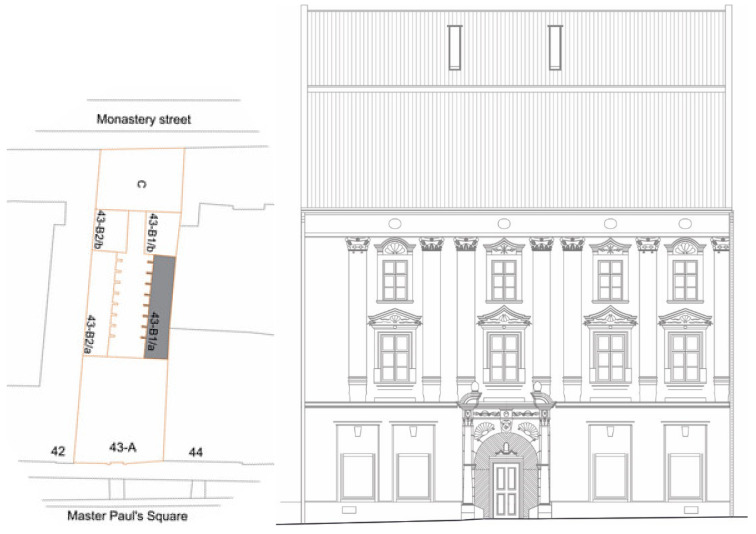
Division of the analyzed building no. 43 into various segments (**left**), contemporary decoration of the main facade—view from Master Paul´s Square (**right**) [36].

The historical burgher house selected for the investigation, with the inventory number 43 and also known as the Mariássy Palace or the patrician house, has a rich history. The building is one of the largest and historically most important burgher houses in Master Paul’s Square, Levoča, which falls under the strictest level of monument protection in Slovakia. The house is segmented into six main parts according to the diagram in Figure 2 [36,37].

**Figure 3:**
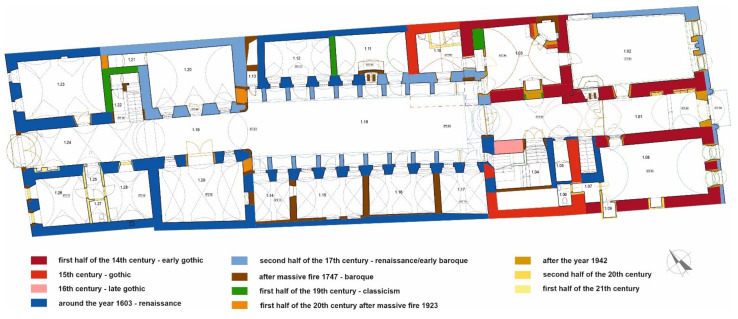
Stylistic analysis and origin of the structures [37].

The oldest building structures were realized in the 14th century. Since that period, the building has passed through a number of renovations and reconstructions. The results of detailed historical research that document the development phases of the building are illustrated in Figure 3.

**Figure 4:**
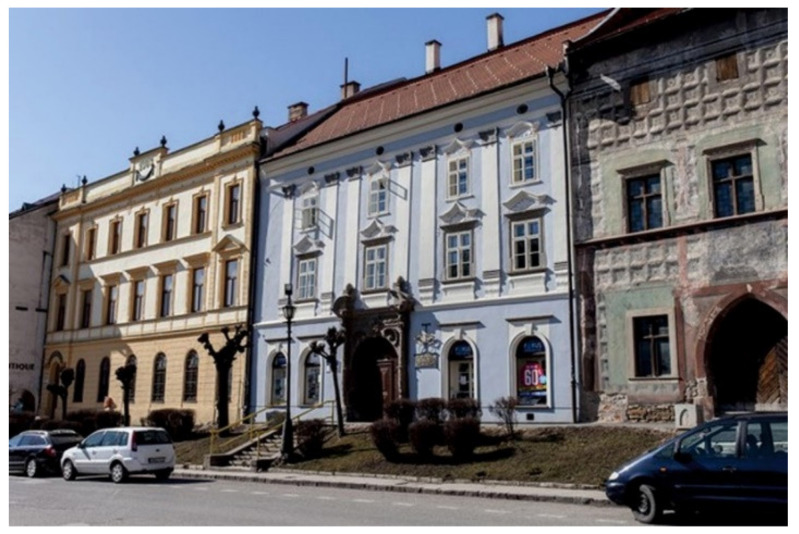
Current state of building no. 43 in Master Paul´s Square, view from the square.

At present, building no. 43, part 43-A hosts the city gallery, part C serves for housing. The atrium parts 43-B1/a, 43-B1/b, 43-B2/a, 43-B2/b are currently unused. The last purpose of this part of the building was mainly for housing and storage space. The current state of building no. 43 in Master Paul´s Square is shown in Figure 4 [36,37].

### 2.2. Sampling

**Figure 5:**
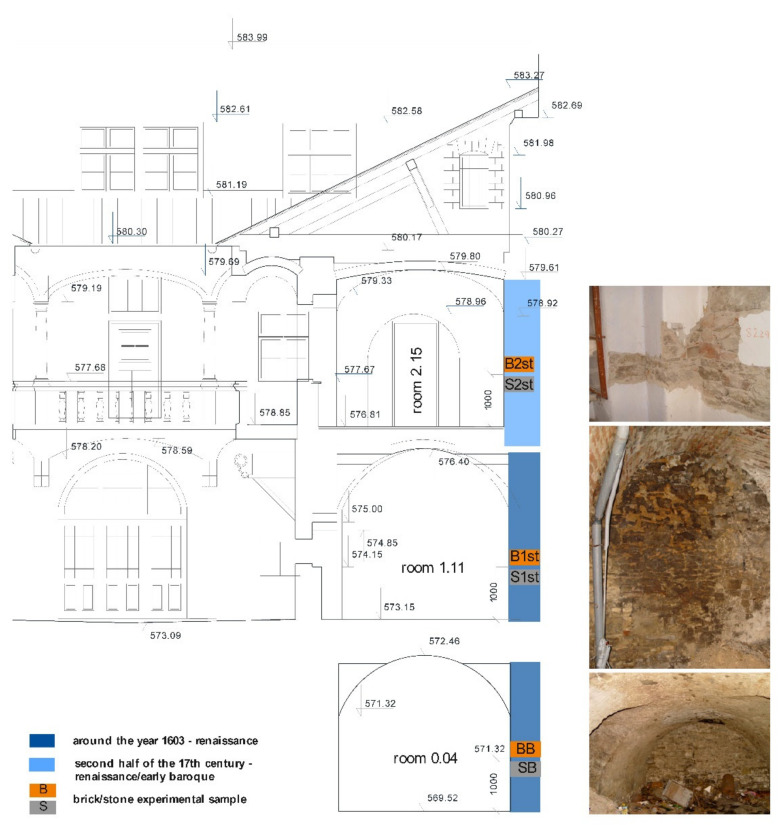
Location of the sampling points in the building.

Three sampling points of building materials for the radioactivity analysis were chosen in the middle part of section 43-B1/a as seen in Figure 2 and Figure 5. Although this was not the oldest part of the building, there were several reasons for choosing this part of the building in terms of authenticity [36,37], e.g., minimal or no changes in room layout during use for approx. 400 years, original or minimally modified building structures with historical materials: masonry, mortars, plasters, this part of the building was not affected by the fires, and the 43-B1/a part of the building includes a basement, so it was possible to take material from all three floors. The stone (S) and brick samples (B) were collected from each sampling point during spring. Indoor air temperature in the rooms was in the interval 19.3–20.6 °C, relative air humidity 57%. All spaces had only natural ventilation and the windows were closed during sampling. Therefore, ventilation was carried out only by infiltration through the old historical wooden double windows.

#### 2.2.1. Basement

The basement in the courtyard at elevation 569.52 m a.s.l. has an entrance through a stone rectangular portal from the courtyard. The space, accessible by stairs, has a floor made of steamed clay and is vaulted with a barrel vault with lunettes; on the south wall there is a ventilation opening to the facade of the square. Samples of building stone (SB) and solid fired brick (BB) were taken from the northeastern wall, the construction of which dates back to around 1600 [36,37]. The sampling points were located about 1000 mm above the floor level.

#### 2.2.2. First Floor

Yard wing 43-B1/a at elevation 573.15 m a.s.l. extends over an area of 84 m^2^. It consists of four rooms being accessible from the courtyard. Room No. 1.11 which was chosen for the sampling is vaulted with a barrel vault with lunette sections [36,37]. Samples of building stone (S1st) and solid fired bricks (B1st) were taken from the northeast wall of the room approximately 1000 mm above the floor level.

#### 2.2.3. Second Floor

The courtyard wing 43-B1/a on the second floor at elevation 576.81 m a.s.l. was built in the early Baroque style [36,37]. It consists of four original rooms. Samples of building stone (S2nd) and solid fired bricks (B2nd) were taken from the northeast wall of room 2.15 approximately 1000 mm above floor level. Before removing the material, a modern lime-cement layer of plasters with painting was removed.

### 2.3. Sample Preparation for the Analysis

The samples were reduced to smaller sizes by a jaw crusher (BRIO BCD 3) and then ground using a planetary rotary mill (SFM-1) to a prescribed particle size of 0.5 mm. Consequently, the powders of material samples were homogenized and dried at 105 °C in a laboratory oven to a constant weight. Subsequently, the bulk samples were homogeneously dispersed into Marinelli type containers (450 mL volume), weighed, and, after closure, subjected to Rn equilibrium for more than 150 days.

### 2.4. Chemical Analysis

The chemical composition of bricks was determined by X-ray fluorescence analysis (XRF) using SPECTRO iQ II (Ametek, Weiterstadt, Germany). Samples were measured in powder form (4 g) for 10 min.

### 2.5. Measurement of Radionuclides’ Activity Concentration

The activity concentrations of radionuclides (^226^Ra, ^232^Th, and ^40^K) in studied materials were measured using gamma ray spectrometry. Measurements were carried out using an EMS-1A SH (Empos, Prague, Czech Republic) detection system equipped with a NaI/Tl scintillation detection probe and an MC4K multichannel analyzer with optimized resolution of 818 V, 4.096 channels, and with 9 cm of lead shielding and internal lining of 2 mm tinned copper.

The specific activity concentrations of ^226^Ra, ^232^Th, and ^40^K were determined in Bq/kg using the count spectra. The ^40^K radionuclide was measured directly through its gamma ray energy peak at 1461 keV, while activities of ^226^Ra and ^232^Th were calculated based on the mean value of their respective decay products. Activity of ^226^Ra was measured using the 351.9 keV gamma rays from ^214^Pb and the activity of ^232^Th was measured using the 238.6 keV gamma rays of ^212^Pb. The same counting time of 86.400 s (24 h) was used for all measured samples. The characteristics of the measured radiation quantities of particular radionuclides are given in Table 1.

### 2.6. Radiological Indices and Doses Due to Building Materials

To assess the collective impact of activity concentrations of the radionuclides in a single quantity, the radiological indices and dose parameters selected are given in Table 2.

The gamma activity concentration *Iγ* has been defined by the European Commission according to Formula (1) [38,39,40]. Alpha index *Iα* (2) [41] was used to estimate exposure due to the radon gas emanation from building materials. Activity utilization index *AUI* was calculated using Formula (3) [42].

(1) $$I\gamma =\frac{ARa}{300\;\mathrm{Bq}/\mathrm{kg}}+\frac{ATh}{200\;\mathrm{Bq}/\mathrm{kg}}+\frac{AK}{3000\;\mathrm{Bq}/\mathrm{kg}}\left(-\right),$$

(2) $$I\alpha =\frac{ARa}{200\;\mathrm{Bq}/\mathrm{kg}}\;\left(-\right),$$

(3) $$\;\;\;\;\;\;AUI=\frac{ARa}{50\;\mathrm{Bq}/\mathrm{kg}}\cdot \;0.0809+\frac{ATh}{50\;\mathrm{Bq}/\mathrm{kg}}\cdot 0.4798+\frac{AK}{500\;\mathrm{Bq}/\mathrm{kg}}\cdot 0.4392\;\left(-\right),$$

where the values 0.00809, 0.4798, and 0.4392 represent fractional percentages of the total dose from ^226^Ra, ^232^Th, and ^40^K (*f_Ra_* = 8.09%, *f_Th_* = 47.98%, and *f_K_* = 43.92%)

The hazard indices were represented by external *H_ex_* and internal *H_in_* hazard risk indices, which are necessary to assess the potential risk resulting from the construction materials used. In principle, the external hazard index *H_ex_* is used to estimate the radiological risk caused by exposure to the external natural radioactive gamma source. The equation is based on the assumption that the activity of 370 Bq/kg of ^226^Ra, 259 Bq/kg of ^232^Th, and 4810 Bq/kg of ^40^K produce the same gamma ray dose rate. *H_ex_* maximum allowable level is expressed based on the corresponding maximum acceptable *Ra_eq_* limit (370 Bq/kg). *Ra_eq_* is expressed by (6) [43,44,45]. Since radon and its short-lived decay products are also hazardous to the respiratory organs, the internal hazard index *H_in_* has been introduced. Both indices were calculated using the expressions (4) and (5) [46,47,48].

(4) $$Hex=\frac{ARa}{370\;\mathrm{Bq}/\mathrm{kg}}+\frac{ATh}{258\;\mathrm{Bq}/\mathrm{kg}}+\frac{AK}{4810\;\mathrm{Bq}/\mathrm{kg}}\left(-\right)$$

(5) $$Hin=\frac{ARa}{180\;\mathrm{Bq}/\mathrm{kg}}+\frac{ATh}{258\;\mathrm{Bq}/\mathrm{kg}}+\frac{AK}{4810\;\mathrm{Bq}/\mathrm{kg}}\left(-\right)$$

(6) $$Raeq=A_{Ra}+1.43A_{Th}+0.077A_{K}\left(\mathrm{Bq}/\mathrm{kg}\right)\;$$

Outdoor external dose *D_out_* was calculated using Formula (7) [49,50]. The European Commission, in 1999 [43], introduced the indoor external dose *D_in_* described by Formula (8). The quantitative coefficients used in *D_out_* and *D_in_* calculations are expressed in nGy/h per 1 Bq/kg. The annual effective dose equivalent is used to estimate the health risk associated with exposure of an individual. The annual effective doses are defined for outdoor, *E_out_* (9), and indoor, *E_in_* (10), exposures [2,51,52]. To estimate the *E_in_* and *E_out_,* the conversion factor (0.7) from absorbed dose rate in air in Sv/Gy to effective dose rate in mSv/yr is used. The occupancy factor represents the proportion of time spent in the indoor and outdoor environments and differs for the outdoor dose *E_out_* (occupancy factor of 0.2) and indoor dose *E_in_* (occupancy factor of 0.8). The excess lifetime cancer risk (*ELCR*) indicator estimates the potential of cancer development over a lifetime, caused by irradiation from building materials. *ELCR* was calculated based upon values of E_out_ and E_in_ using Formulas (11) and (12), where *LE* is life expectancy (70 years) [2,53] and *RF* is fatal risk factor per Sievert, which is 0.05 according to [54]. The annual dose equivalent of gonads *AGDE* due to the specific activities of ^226^Ra, ^232^Th, and ^40^K was estimated using Formula (13) [51,55]. The annual effective dose equivalent represents the degree of genetic significance of the annual dose that the reproductive organs of a population receive. Organs with rapidly dividing cells, such as gonads, active bone marrow cells, lungs, testes, ovaries, and bone surface cells, are considered interesting by the UN Scientific Committee on the Effects of Atomic Radiation. The effective dose rate *D_organs_* delivered to a particular organ can be calculated using the relation in (14) [56], where f is the conversion factor of organ dose from air dose. The conversion factor for lungs, ovaries, bone marrow, testes, and the whole body are 0.64, 0.58, 0.69, 0.82, 0.68, respectively [39].

(7) $$D_{out}=0.436A_{Ra}+0.599A_{Th}+0.0417A_{K}\left(\mathrm{nGy}/h\right)$$

(8) $$D_{in}=0.92A_{Ra}+1.1A_{Th}+0.081A_{K}\;\;\;\left(\mathrm{nGy}/h\right)$$

(9) $$E_{out}=D_{out}\times 0.2\times 8760\;\left(h\right)\times 0.7\left(\mathrm{mSv}/y\right)$$

(10) $$E_{in}=D_{in}\times 0.8\times 8760\;\left(h\right)\times 0.7\left(\mathrm{mSv}/y\right)$$

(11) $$ELCR_{out}=E_{out\;}\times LE\times RF\;\;\;\;\;\;\;\left(-\right)$$

(12) $$ELCR_{in}=E_{in\;}\times LE\times RF\;\;\;\;\;\;\left(-\right)$$

(13) $$AGDE=3.09\;A_{Ra}+4.18\;A_{Th}+0.314\;A_{K}\;(\mu \mathrm{Sv}/y)$$

(14) $$D_{organs}=E_{in\;}\times f\;$$

## 3. Results and Discussion

### 3.1. Chemical Composition of Samples

The chemical analysis of the collected materials, measured by an X-ray fluorescence analyzer (XRF) (Ametek, Germany), are given in Table 3.

The chemical composition of brick samples in Table 3 corresponds to the usual contents of the main elements in brick clay [57]. According to the chemical composition of the stone samples, the stones could probably belong to granite type [58]. However, the chemical composition of the stone samples in the basement differs slightly from those collected from the 1st and 2nd floor. To compare the similarity of the samples, the expression of the concentration ratio of CaO to SiO_2_ is commonly used. While for brick samples the CaO/SiO_2_ concentration ratio varies in a relatively narrow interval of 15–20, in the case of stone samples the differences are significantly larger. The CaO/SiO_2_ ratio of stone samples from the basement reached the value of 26.8, which is several times higher than the CaO/SiO_2_ ratio of stone samples from the 1st and 2nd floor (4.9 and 2.6, respectively). This finding points to non-identical stone materials analyzed.

Granite, widely used as a cladding on city buildings and also architecturally in homes, contains an average of 3 ppm (40 Bq/kg) uranium and 17 ppm (70 Bq/kg) thorium [59]. Based on the contents of natural potassium, uranium, and thorium, the activity of radioactive isotopes should be estimated in particular samples. As seen in Table 1, higher activity concentrations of ^40^K can be expected in brick samples since the total potassium content in bricks was higher than in stone samples. Content of 1% of natural potassium in rock minerals corresponds to 313 Bq/kg of ^40^K radionuclide as reported in [60]. It is assumed that ^40^K concentrations could range from 376 to 761 Bq/kg for the individual samples analyzed. Radium and thorium radioisotope concentrations are difficult to estimate as the measured XRF concentrations are below the detection limit.

### 3.2. Activity Concentrations of Radionuclides

**Figure 6:**
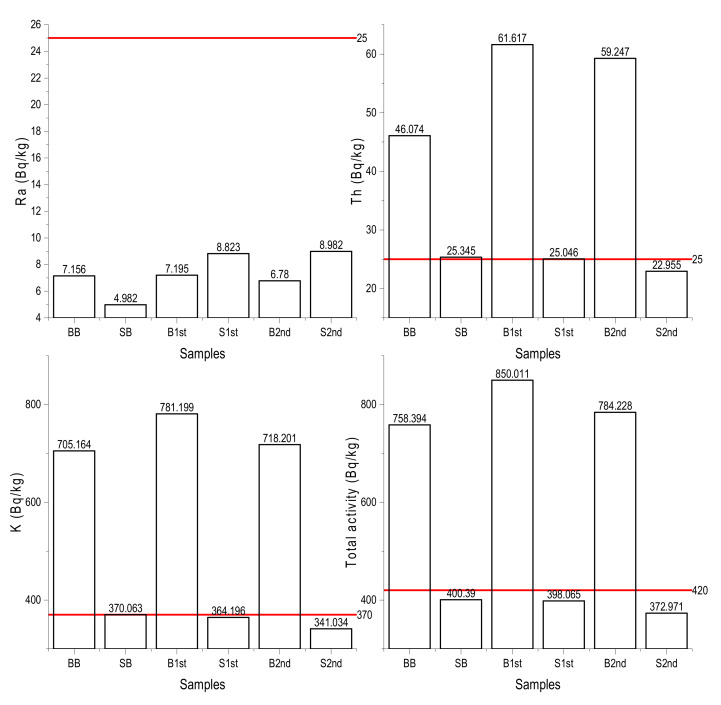
Comparison of measured activity concentrations of ^226^Ra, ^232^Th, and ^40^K in building material samples with the world average concentrations (represented by red lines).

The measured activity concentration of ^226^Ra, ^232^Th, and ^40^K radionuclides in the stone and brick samples varied from 6.78 ± 2.1 to 8.98 ± 1.8, 22.96 ± 7.3 to 61.62 ± 7.6, 341.04± 10.2 to 781.19 ± 10.9 Bq/kg, respectively, as shown in Table 4. Profiles of the activity concentration of various radionuclides in building material samples and world averages are shown in Figure 5. The total activity concentrations varied from 372.97 to 850.01 Bq/kg. The activity concentration of ^226^Ra for bricks and stones and activity concentrations of ^232^Th and ^40^K for stone samples were lower than the world average concentrations of these radionuclides in building materials that are 25, 25, and 370 Bq/kg, respectively, as per [61]. The values of stone samples radioactivity were lower than in Gupta, 2011 [62], likewise for brick activity concentrations compared to Turhan, 2008 [63] and Raghu, 2016 [64]. The average of total activity concentration (594.01 Bq/kg) expressed as sum of ^226^Ra and ^232^Th and ^40^K concentrations is higher than the world average of total activity concentration of these radionuclides in building material samples (420 Bq/kg). The total activity concentrations of ^226^Ra, ^232^Th, and ^40^K of building material samples are shown in Figure 6.

As assumed, the measured concentrations of ^40^K were almost twice as high in brick samples than in stone samples.

**Figure 7:**
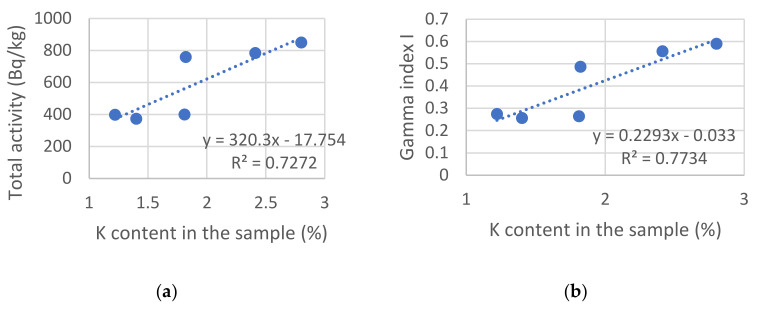
Correlation between the potassium content and the radiological parameters: (**a**) total activity of radionuclides; (**b**) gamma index of radionuclides.

A correlation was found between the chemical composition, specifically the potassium content in the building material samples and the total activity of radionuclides (Figure 7a) with the correlation coefficient R = 0.85, as well as the dependence between the gamma index and the potassium content (Figure 7b) with a correlation coefficient of R = 0.88. The correlation was even more significant for the brick materials themselves.

**Figure 8:**
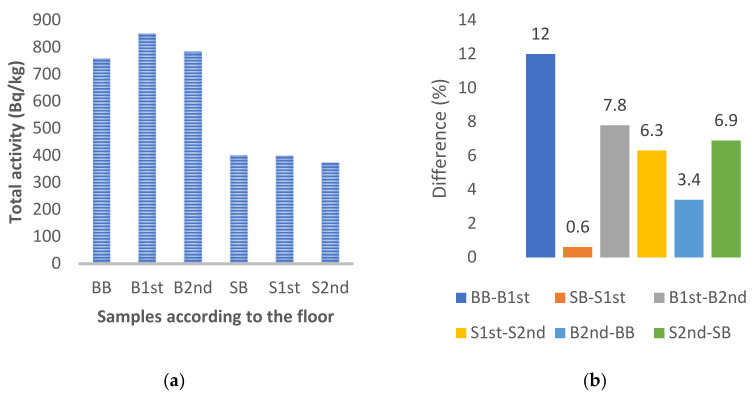
Comparison of the effect of the sample’s floor to total activity of radionuclides (**a**); the differences in total activities of radionuclides in samples collected from different floors (**b**).

The measured activity concentrations of samples were also analyzed in terms of the samples’ location from the basement to the second floor. No fundamental difference was detected in the radioactivity of the materials taken from the individual floors. The radioactivity of the materials of the stone samples slightly decreased with increasing floor as can be seen in Figure 8a. This was not observed for the brick samples. The differences between the particular values of total activities per samples collected from the basement, 1^st^, and 2nd floor ranged from 0.6 to 12% (Figure 8b).

### 3.3. Estimation of External Gamma Exposure Due to Building Materials

**Figure 9:**
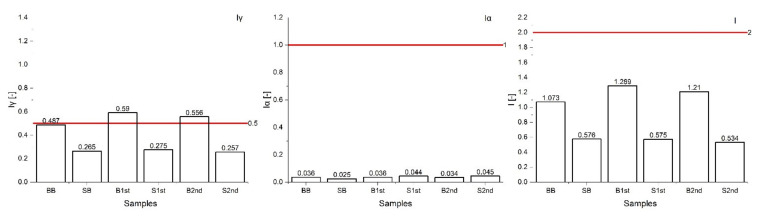
Comparison of the level indices *Iγ, Iα*, and *AUI* with the limit values (represented by red lines).

**Figure 10:**
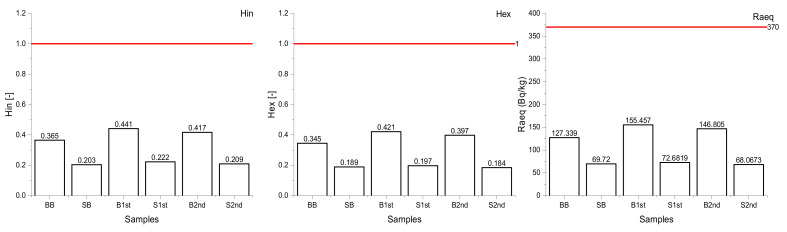
Comparison of the hazard indices and *Ra_eq_* with the limit values (represented by red lines).

**Figure 11:**
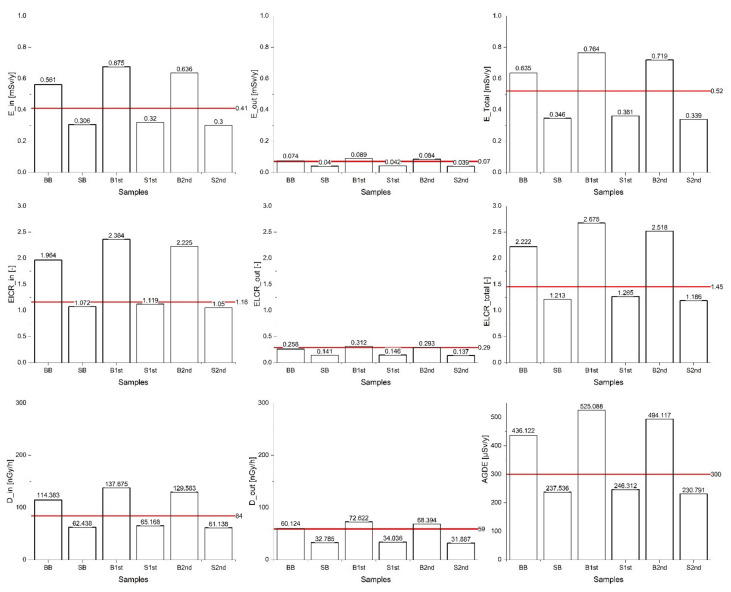
Comparison of the dose parameters with the world average values (represented by red lines).

**Figure 12:**
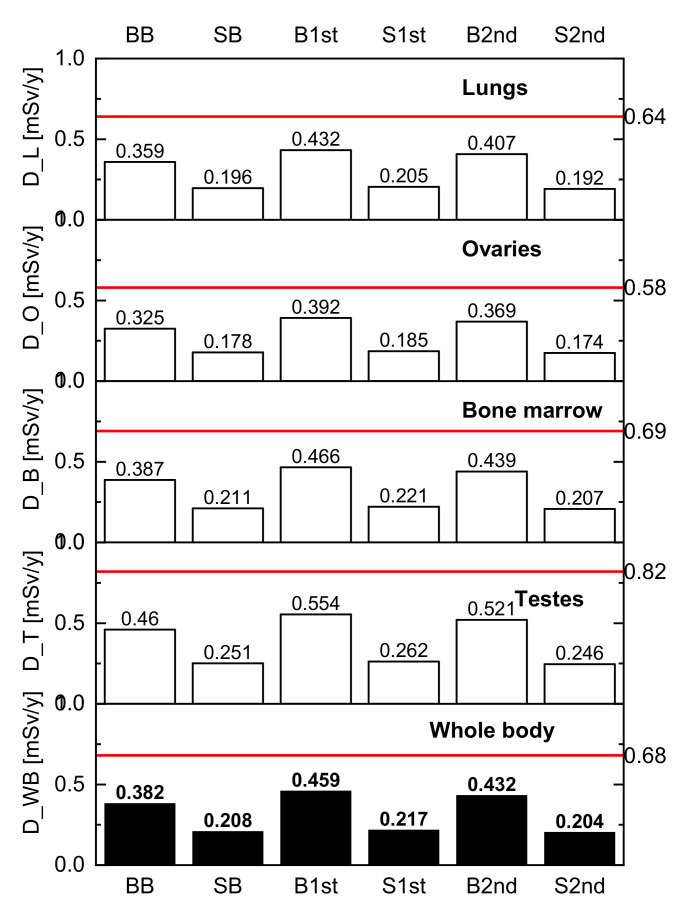
Absorbed dose by different organs and the particular limit values (represented by red lines).

Potential radiological risk of the bricks and stones from a historical building in Levoča was estimated on the basis of calculated radiological indicators and doses given in the following Figure 9, Figure 10, Figure 11 and Figure 12.

The calculated gamma indices, *Iγ,* observed for the samples, ranged in an interval of 0.26–0.60, not exceeding the restricted limit for bulk materials *Iγ* = 1. However, when considering the recommended unrestricted value *Iγ* = 0.5 which corresponds to the annual dose of 0.3 mSv/year, 33% of samples did not meet this dose criterion (Figure 9). According to activity concentration index (ACI) approach, the materials having the *Iγ* in the interval 0.5–1 can be classified for restricted use, e.g., in bridges or roads or only for low-occupancy buildings. The highest value was found for the B1st sample (*Iγ* = 0.590). The gamma indices for bricks were higher compared to Amiri et al., 2014 [65], who reported six times lower *Iγ* found for bricks, and two times higher than in Lima, 2015 [53].

Alpha index *Iα* was in the range 0.03 to 0.05 (Figure 9). Iα should be lower than permissible value (*Iα* = 1) which relates to 200 Bq/kg. Materials with ^226^Ra concentration lower than 200 Bq/kg cannot cause indoor radon activity higher than 200 Bq/kg, therefore, it can be assumed that building materials in this study will not pose a risk to exceeding the limit value of Rn emanation from building materials. The *Iα* values were similar compared to [66] but lower compared to [67]. Values of Iα were lower for stones and higher for brick samples compared to Lyngkhoi, 2020 [67]. Activity utilization indices *AUI* ranged from 0.53 to 1.29, and were less than the recommended value (I ≤ 2). The AUI values in this study were comparable with those in the study by Ademila, 2020 [68].

Both *H**_in_*** and *H_ex_* risk indicators of all samples studied were lower than 1. These results correspond to the studies by Fares, 2019 [69] and Tuo, 2020 [70] for bricks and Ajayi, 2013 [71] for stones. The total gamma output from the combination of ^226^Ra, ^232^Th, and ^40^K radionuclide activities represented through the radium equivalent activity indicator (*Ra_eq_*) was under the limit value (370 Bq/kg), ranging from 68.067 to 155.459 Bq/kg (Figure 10).

The calculated dose parameters resulting from the activity concentrations of measured radionuclides are presented and compared to the world average values in Figure 11. Outdoor external dose *D_out_* ranged from 31.887 to 72.621 nGy/h. The values linked to brick samples were found to be higher than the world average value of 59 nGy/h. The values of calculated indoor external dose *D_in_* ranged from 61.138 to 137.675 nGy/h with an average 95.06 nGy/h, which is 1.13 times more than the world average of 84 nGy/h as per [2].

The value of the annual indoor effective dose *E_in_* ranged from 0.30 to 0.68 mSv/y, and in the case of the brick samples, exceeded the world average of 0.41 mSv/y (Figure 11). The annual outdoor effective dose *E_out_* for the building material samples was identified in the interval 0.04 to 0.09 mSv/y, and, similarly to *E_in_,* was higher than the world average of 0.07 mSv/y for the brick samples. These findings correspond to the results reported by [72]. The total annual effective dose *E_total_* (0.53 mSv/y) was similar to the world average value (0.52 mSv/y) and does not exceed the criterion limit of 1 mSv/y as per ICRP-60 [54]. However, the total annual effective dose *E_total_* for brick samples was significantly higher than that of the stone samples.

The *ELCR_total_* for outdoor and indoor exposure reached values from 1.05 to 2.36 with an average of 1.85, which is 27% higher than the world average (1.45). However, the results in this study are lower compared to [73], who reported the value of 3.21.

The calculated annual gonadal equivalent dose *AGDE* connected to the samples ranged in an interval of 230.97–525.09 µSv/y. The world average of *AGDE* for houses was reported to be about 370 mSv/yr whereas the standard UNSCEAR value for *AGDE* is 300 mSv/yr. Darwish [66] presents the value of 520 µSv/y in stone houses. The highest value was found for the B1st sample. The *AGDE* values for bricks exceeded the world average value reported in an OECD report [26]. *AGDE* results for the samples were double those for stone and four times higher for bricks than reported in [67]. The application of waste materials as replacement for cement in the production of concrete could represent another risk of increasing the activity of radionuclides and subsequently their negative effects on the human body [74].

Figure 12 shows the estimation of the absorbed dose by individual organs. The highest sensitivity has been proved in the case of testes and the lowest for ovaries. These results are a little bit lower compared to [75]. However, it should be noted here that some experts do not recommend using low values of radionuclides’ activity for cancer risk estimation. At the same time, the authors of the present study would like to point out that the results refer only to the study of external exposure due to built-in materials and not to the assessment of the radon levels.

## 4. Conclusions

The paper presents a radioactivity study on building materials (stone and bricks) of a historical building in the city of Levoča, Slovakia. The research was aimed at examining the possible risks originating from specific activities of radionuclides in building materials in the building after reconstruction. Building materials of three floors—basement, 1st floor, and 2nd floor—show some variation in activity concentration of three radionuclides (^226^Ra, ^232^Th, and ^40^K) from site to site. The average measured activity concentrations of radionuclides were 7.32 Bq/kg for ^226^Ra, 40.05 Bq/kg for ^232^Th, and 546.64 Bq/kg for ^40^K radionuclides. The average total activity concentration in building materials of 594.1 Bq/kg exceeded the world average value (420 Bq/kg).

The potential outdoor and indoor risks were evaluated through the various radiological indices and estimated doses based on the measured activity concentrations of ^226^Ra, ^232^Th, and ^40^K radionuclides in the samples. The calculated gamma indices, *Iγ,* ranged from 0.26 to 0.60, not exceeding the EU limit for bulk materials *Iγ* = 1. Radiation values slightly higher than world average were noticed in the case of the outdoor external dose and outdoor annual effective dose and indoor annual effective dose which were identified to be 13 and 14% higher, respectively, than the average world values. Total annual effective dose of 0.53 mSv/y was almost identical to the average world value (0.52 mSv/y). Exceeding of the average world values was also observed for the total ELCR (by 27%) and for the annual gonadal dose equivalent (by 21%).

The results obtained in this study confirmed that bricks are responsible for a higher level of natural radiation than the used natural stone material. The radiological parameters related to bricks were more than double compared to building stone.

The observed strong correlation between the potassium content in the building materials, measured by XRF, and total activity concentration of radionuclides (R = 0.85) confirm the possibility to estimate the radioactivity of brick and stone materials based on chemical analysis. The results of this case study underline that, based on the chemical analysis and determination of the total potassium content, it is possible not only to estimate the activity concentrations of 40 K but also the total activity of radionuclides. During the reconstruction of buildings, external gamma radiation should be controlled in relation to the building materials used. The chemical analysis of materials, which can be performed in a shorter time frame than the determination of activity concentrations of radionuclides, which takes more than 40 days, could serve as input screening information on the need for detection of activity of radionuclides.

Although some radiological parameters were higher than the world average, no exceeding of the established limits for gamma index and annual dose was recorded. Thus, the analyzed materials cannot pose any significant risk in a long-term horizon and therefore the building could be recommended for residential purposes after the reconstruction. Since only external exposure to gamma radiation was evaluated in this study, further radon monitoring should be performed to confirm the safety of the indoor spaces considered for residential purposes.

## Figures and Tables

**Table 1:** Measured radiation quantities and their characterization.

Radiation Quantities	Labeling	Unit	Characteristic
Activity concentration of ^226^Ra	*A_Ra_*	Bq/kg	the activity of ^226^Ra in 1 kg of the analyzed material, measured as the number of spontaneous nuclear transformations of the ^226^Ra radionuclide per second
Activity concentration of ^232^Th	*A_Th_*	Bq/kg	the activity of ^232^Th in 1 kg of the analyzed material, measured as the number of spontaneous nuclear transformations of the ^232^Th radionuclide per second
Activity concentration of ^40^K	*A_K_*	Bq/kg	the activity of ^40^K in 1 kg of the analyzed material, measured as the number of spontaneous nuclear transformations of the ^40^K radionuclide per second

**Table 2:** Radiological parameters and their characterization.

Radiological Indices and Doses	Labeling	Unit	Characteristic
Gamma activity concentration index	*Iγ*	*-*	estimation of the gamma radiation hazard associated with the radionuclides inside of the building materials, calculated from activity concentration measurements of the material
Alpha index	*Iα*	*-*	estimation of exposure due to the radon gas emanation from building materials, calculated from activity concentration measurements of the material
Activity utilization index	*AUI*	*-*	estimation of total dose rates in air from naturally occurring radionuclides in building materials, calculated from activity concentration measurements of the material
Radium equivalent activity	*Ra_eq_*	Bq/kg	expression of the specific activities of ^226^Ra, ^232^Th, and ^40^K by a single quantity, which takes into account the radiation hazards associated with radon and its progeny
Internal hazard index	*H_in_*	-	index to control the hazard due to inhalation of alpha particles emitted from the short-lived radionuclides in buildings
External hazard index	*H_ex_*	-	obtained from *Ra_eq_* expression through the supposition that its maximum value allowed (equal to unity) corresponds to the upper limit of *Ra_eq_* (370 Bq/kg)
Indoor external dose	*D_in_*	nGy/h	the total absorbed gamma dose rate indoors at 1 m above the ground
Outdoor external dose	*D_out_*	nGy/h	the total absorbed gamma dose rate outdoors at 1 m above the ground
Indoor effective dose	*E_in_*	mSv/y	annual dose indoors considering the conversion factor for environmental exposure to gamma rays
Outdoor effective dose	*E_out_*	mSv/y	annual dose outdoors considering the conversion factor for environmental exposure to gamma rays
Excess lifetime cancer risk	*ELCR*	-	estimation of the potential of cancer development over a lifetime, caused by irradiation from building materials
Effective dose rate to different body organs	*D_organ_*	mSv/y	the mean energy absorbed per unit mass averaged over the entire tissue or organ
Annual gonadal dose equivalent	*AGDE*	µSv/y	evaluation fo the potential effects of the specific activities of ^226^Ra, ^232^Th, and ^40^K on certain important organs, such as reproductive organs (gonads), bone marrow, and bone cells

**Table 3:** Chemical analysis of stone and brick samples—main components.

Samples	SiO_2_	CaO	Al_2_O_3_	MgO	P_2_O_5_	SO_3_	Fe_2_O_3_	K	Th	U
	%								ppm	ppm
BB	39.06	2.06	9.22	1.92	0.23	0.11	3.10	1.82	7.1	˂3
SB	38.80	1.45	9.20	1.77	0.27	0.07	3.23	1.81	3.2	˂3
B1st	40.29	2.00	10.96	1.96	0.13	0.10	4.33	2.08	˂1.8	˂3
S1st	28.06	5.75	6.83	2.03	0.08	0.34	2.31	1.22	˂2.0	˂3
B2nd	41.69	2.25	11.45	2.05	0.14	0.07	4.63	2.41	˂2.0	˂3
S2nd	29.13	11.32	7.02	2.21	0.15	0.05	2.81	1.40	˂2.0	˂3

**Table 4:** Values of radionuclide activity concentrations in samples.

**Samples**	**^226^** **Ra**	**^228^** **Th**	**^40^** **K**
**Bq/kg**			
BB	7.16 ± 2.1	46.07 ± 7.1	705.16 ± 10.1
SB	4.98 ± 2.2	25.35 ± 7.7	370.06 ± 10.6
B1st	7.19 ± 1.9	61.62 ± 7.6	781.19 ± 10.9
S1st	8.82 ± 2.0	25.05 ± 7.2	364.16 ± 10.3
B2nd	6.78 ± 2.1	59.25 ± 7.5	718.21 ± 10.8
S2nd	8.98 ± 1.8	22.96 ± 7.3	341.04 ± 10.2
Average	7.32	40.05	546.64

## Data Availability

Data available on request from the authors.

## References

[1] International Atomic Energy Agency Naturally occurring radioactive material (NORM V). Proceedings of the International Symposium on Naturally Occurring Radioactive Material.

[2] United Nations Scientific Committee on the Effects of Atomic Radiation (UNSCEAR) (2000). Sources, Effects and Risks of Ionizing Radiation.

[3] Ramachandran T.V. (2011). Background radiation, people and the environment Iran. J. Radiat. Res..

[4] Suliman I.I., Alsafi K. (2021). Radiological Risk to Human and Non-Human Biota Due to Radioactivity in Coastal Sand and Marine Sediments, Gulf of Oman. Life.

[5] Lasheen E.S.R., Rashwan M.A., Osman H., Alamri S., Khandaker M.U., Hanfi M.Y. (2021). Radiological Hazard Evaluation of Some Egyptian Magmatic Rocks Used as Ornamental Stone: Petrography and Natural Radioactivity. Materials.

[6] Jasaitis D., Pečiulienė M. (2021). Natural Radioactivity and Radon Exhalation from Building Materials in Underground Parking Lots. Appl. Sci..

[7] Singovszka E., Estokova A. (2019). Evaulation of potential radiation hazard in a historical building in Košice, Slovakia. Advances and Trends in Engieneering Sciences and Technologies III.

[8] Miklyaev P.S., Petrova T.B., Shchitov D.V., Sidyakin P.A., Murzabekov M.A., Tsebro D.N., Marennyy A.M., Nefedov N.A., Gavriliev S.G. (2022). Radon transport in permeable geological environments. Sci. Total Environ..

[9] Kocsis E., Tóth-Bodrogi E., Peka A., Adelikhah M., Kovács T. (2021). Radiological impact assessment of different building material additives. J. Radioanal. Nucl. Chem. Artic..

[10] Manić G., Manić V., Nikezić D., Krstić D. (2015). The dose of gamma radiation from building materials and soil. Nukleonika.

[11] United Nations Scientific Committee on the Effects of Atomic Radiation (UNSCEAR) (2010). Report of the United Nations Scientific Committee on the Effects of Atomic Radiation 2010.

[12] Shahrokhi A., Adelikhah M., Chalupnik S., Kocsis E., Toth-Bodrogi E., Kovács T. (2020). Radioactivity of building materials in Mahallat, Iran—An area exposed to a high level of natural background radiation—Attenuation of external radiation doses. Mater. De Construcción.

[13] Dodge-Wan D., Mohan Viswanathan P. (2021). Terrestrial gamma radiation dose rate mapping and influence of building materials: Case study at Curtin University campus (Miri, Sarawak, Malaysia). J. Radioanal. Nucl. Chem. Artic..

[14] European Commission (1999). Radiation Protection 112. Radiological Protection Principles Concerning the Natural Radioactivity of Building Materials, Directorate General Environment, Nuclear Safety and Civil Protection.

[15] Mas J.L., Caro Ramírez J.R., Hurtado Bermúdez S., Leiva Fernández C. (2021). Assessment of natural radioactivity levels and radiation exposure in new building materials in Spain. Radiat. Prot. Dosim..

[16] Belgin E.E., Aycik G. (2015). ^226^Ra, ^232^Th and ^40^K Activity concentrations and radiological hazards of building materials in Mugla, Turkey. J. Sci. Technol..

[17] Lima M., Sanjurjo-Sánchez J., Alves C. (2017). Assessment by Portable Gamma Spectrometry of External Gamma Radiation Hazard due to Granitic Materials and Indoor Space Typology. Geosciences.

[18] Yang Y.X., Wu X.M., Jiang Z.Y., Wang W.X., Lu J.G., Lin J., Wang L.M., Hsia Y.F. (2005). Radioactivity concentrations in soils of the Xiazhuang granite area, China. Appl. Radiat. Isot..

[19] Turhan S., Kurnaz A., Karataşlı M. (2022). Evaluation of natural radioactivity levels and potential radiological hazards of common building materials utilized in Mediterranean region, Turkey. Environ. Sci. Pollut. Res..

[20] Trevisi R., Leonardi F., Risica S., Nuccetelli C. (2018). Updated database on natural radioactivity in building materials in Europe. J. Environ. Radioact..

[21] Sabbarese C., Ambrosino F., D’Onofrio A., Roca V. (2021). Radiological characterization of natural building materials from the Campania region (Southern Italy). Constr. Build. Mater..

[22] Al-Zahrani J.H. (2017). Estimation of natural radioactivity in local and imported polished granite used as building materials in Saudi Arabia. J. Radiat. Res. Appl. Sci..

[23] Yu K.N., Guan Z.J., Cheung T., Cheung T.T.K., Lo T.Y. (2000). Light weight concrete: ^226^Ra, ^232^Th, ^40^K contents and dose reduction assessment. Appl. Radiat. Isot..

[24] Estokova A., Palascakova L. (2013). Assessment of natural radioactivity levels of cements and cement composites in the Slovak Republic. Int. J. Environ. Res. Public Health.

[25] Bavarnegin E., Moghaddam M.V., Fathabadi N. (2013). Natural radionuclide and radiological assessment of building materials in high background radiation areas of Ramsar, Iran. J. Med. Phys..

[26] Beyala Ateba J.F., Owono Ateba P., Simo A., Ben-Bolie G.H., Ekobena H.F., Abega C.R., Mvondo S. (2017). Estimation of radiation hazard indices from syenite building rocks in the South-western region of Cameroon. Radioprotection.

[27] Penabei S., Bongue D., Maleka P., Dlamini T., Saïdou, Shouop C.G., Halawlaw Y., Ebongue A.N., Njock M.K. (2018). Assessment of natural radioactivity levels and the associated radiological hazards in some building materials from Mayo-Kebbi region, Chad. Radioprotection.

[28] Saleh H.M., Salman A.A., Faheim A.A., El-Sayed A.M. (2020). Sustainable composite of improved lightweight concrete from cement kiln dust with grated poly (styrene). J. Clean. Prod..

[29] Saleh H.M., Salman A.A., Faheim A.A., El-Sayed A.M. (2021). Influence of aggressive environmental impacts on clean, lightweight bricks made from cement kiln dust and grated polystyrene. Case Stud. Constr. Mater..

[30] Stevulova N., Vaclavik V., Junak J., Grul R., Bacikova M. (2008). Utilization possibilities of selected waste kinds in building materials preparing. Int. Multidiscip. Sci. GeoConference.

[31] Gola L., Václavík V., Valíček J., Harničárová M., Kušnerová M., Dvorský T., Öchsner A., Altenbach H. (2015). Drainage Concrete Based on Cement Composite and Industrial Waste. Mechanical and Materials Engineering of Modern Structure and Component Design.

[32] Sanjuán M.A., Pacheco-Torgal F., Falkinham J.O., Gałaj J.A. (2022). 9—Coal bottom ash natural radioactivity in building materials. Advances in the Toxicity of Construction and Building Materials.

[33] Sowińska-Heim J. (2020). Adaptive Reuse of Architectural Heritage and Its Role in the Post-Disaster Reconstruction of Urban Identity: Post-Communist Łódź. Sustainability.

[34] Beir V. (1990). Health Effects of Exposure to Low Levels of Ionizing Radiation.

[35] Singovszka E., Estokova A. (2020). Natural Radioactivity of Bricks in Historical Buildings in Slovakia. Int. J. Eng. Res. Afr..

[36] Chalupecký I. (1975). History of Levoca City II.

[37] Janovská M. (2020). Town House No. 43 in Majster Pavol Square in Levoča, Architectural, Historical and Artistic. Historical Research.

[38] NEA-OECD (1979). Nuclear Energy Agency Exposure to Radiation from Natural Radioactivity in Building Materials.

[39] El-Gamal A., Nasr S., El-Taher A. (2007). Study of the spatial distribution of natural radioactivity in the upper Egypt Nile River sediments. Radiat. Meas..

[40] El-Galy M.M., El Mezayn A.M., Said A.F., El Mowafy A.A., Mohamed M.S. (2008). Distribution and environmental impacts of some radionuclides in sedimentary rocks at Wadi Naseib area, southwest Sinai, Egypt. J. Environ. Radioact..

[41] Buranurak S., Pangza K. (2018). Assessment of natural radioactivity levels and radiation hazards of Thai Portland cement brands using Gamma spectrometry technique. Mater. Today Proc..

[42] UNSCEAR (1993). Exposure from Natural Sources of Radiation.

[43] Maxwell O., Wagiran H., Zaidi E., Joel E.S., Tenebe I.T., Oha I.A., Onwuka O.S. (2016). Radiotoxicity risks of radium-226 (226Ra) on groundwater-based drinking at Dawaki, Kuje, Giri and Sabon-Lugbe area of Abuja, North Central Nigeria. Environ. Earth Sci..

[44] Gbenu S.T., Oladejo O.F., Alayande O., Olukotun S.F., Fasasi M.K., Balogun F.A. (2015). Assessment of radiological hazard of quarry products from southwest Nigeria. J. Radiat. Res. Appl. Sci..

[45] Umesha Reddy K., Ningappa C., Sannappa J. (2017). Natural radioactivity level in soils around Kolar Gold Fields, Kolar district, Karnataka, India. J. Radioanal. Nucl. Chem. Artic..

[46] Beck H.L. (1980). Exposure Rate Conversion Factors for Radionuclides Deposited on the Ground.

[47] Saito K., Jacob P. (1995). Gamma ray fields in the air due to sources in the ground. Radiat. Prot. Dosim..

[48] Clouvas A., Xanthos S., Antonopoulos-Domis M., Silva J. (2000). Monte Carlo calculation of dose rate conversion factors for external exposure to photon emitters in soils. Health Physics..

[49] Quindos L.S., Fernandez P.L., Rodenas C., Gomez-Arozamena J., Arteche J. (2004). Conversion factors for external gamma dose derived from natural radionuclides in soils. J. Environ. Radioact..

[50] Shittu A., Aznan Fazli I., Supian S. (2019). Determination of indoor doses and excess lifetime cancer risks caused by building materials containing natural radionuclides in Malaysia. Nucl. Eng. Technol..

[51] Ravisankar R., Vanasundari K., Chandrasekaran V., Suganya M., Vijayagopal P. (2012). Measurement of Natural radioactivity in building materials of Namakkal, Tamilnadu, India using gamma ray spectrometry. Appl. Radiat. Isot..

[52] Diab H.M., Nouh S.A., Hamdy A., El-Fiki S.A. (2008). Evaluation of natural radioactivity in a cultivated area around a fertilizer factory. Nucl. Radiat. Phys..

[53] Lima M., Alves C., Sanjurjo J. (2015). Gamma radiation in rocks used as building materials: The braga granite (Nw Portugal). Cadernos Lab. Xeolóxico de Laxe Coruña.

[54] ICRP (1991). Dose Limits and Risks (Chapter 4). ICRP Publication 60. 1990 Recommendations of the International Commission on Radiological Protection. Ann. ICRP.

[55] El-Taher A. (2010). Gamma spectroscopic analysis and associated radiation hazards of building materials used in Egypt. Radiat. Prot. Dosim..

[56] O’Brien K., Sanna R. (1976). The distribution of absorbed dose-rates in humans from exposure to environmental gamma rays. Health Phys..

[57] Šveda M., Sokolar R., Janik B., Štefunková Z. (2017). Reducing CO_2_ Emissions in the Production of Porous Fired Clay Bricks. J. Mater. Sci..

[58] Behbahania H., Ziaria H., Kambooziaa N., Mansour Khakia A., Mirabdolazimi S.M. (2015). Evaluation of performance and moisture sensitivity of glasphaltmixtures modified with nanotechnology zycosoil as an anti-stripping additive. Constr. Build. Mater..

[59] Zohuri B. (2020). Nuclear fuel cycle and decommissioning. Nuclear Reactor Technology Development and Utilization.

[60] Erdi-Krausz G., Matolin M., Minty B., Nicolet J.P., Reford W.S., Schetselaar E.M. (2003). Guidelines for Radioelement Mapping Using Gamma Ray Spectrometry Data.

[61] United Nations Scientific Committee on Effects of Atomic Radiation (UNSCEAR) (1988). Sources Effects and Risks of Ionizing Radiation.

[62] Gupta M., Chauhan R.P. (2011). Estimating radiation dose from building materials. Iran. J. Radiat. Res..

[63] Turhan S., Baykan U.N., Sen K. (2008). Measurement of the natural radioactivity in building materials used in Ankara and assessment of external doses. J. Radiol. Prot..

[64] Raghu Y., Ravisankar R., Chandrasekaran A., Vijayagopal P., Venkatraman B. (2016). Assessment of Natural Radioactivity and Radiological Hazards in Brick Samples Used in Tiruvannamalali District, Tamilnadu, India, with a Statistical Approach. Health Phys..

[65] Amiri J., Shirmardi S.P., Pirayesh Eslamian J. (2014). Measuring natural radioactivity of bricks used in the constructions of Tehran. Arch. Adv. Biosci..

[66] Darwish D.A.E., Abul-Nasr K.T.M., El-Khayatt A.M. (2015). The assessment of natural radioactivity and its associated radiological hazards and dose parameters in granite samples from South Sinai, Egypt. J. Radiat. Res. Appl. Sci..

[67] Lyngkhoi B., Nongkynrih P. (2020). Radioactivity in building materials and assessment of risk of human exposure in the East Khasi Hills District, Meghalaya, India. Egypt. J. Basic Appl. Sci..

[68] Ademila O. (2020). Evaluation of Rock Radiation Hazards for Construction Applications in Parts of Southwestern Nigeria. J. Sci. Res..

[69] Fares S.S. (2019). Natural radioactivity measurement of bricks used in the building materials of Egypt. Arab. J. Nucl. Sci. Appl..

[70] Tuo F., Peng X., Zhou Q., Zhang J. (2020). Assessement of natural radioactivity levels and radiological hazards in building materials. Radiat. Prot. Dosim..

[71] Ajayi O.J., Jere P. (2013). Bashiru, B.B. Assessment of Radiological Hazard Indices of Building Materials in Ogbomoso, South-West Nigeria. Environ. Nat. Resour. Res..

[72] Al-Jundi J., Salah W., Bawa’aneh M.S., Afaneh F. (2006). Exposure to radiation from the natural radioactivity in Jordanian building materials. Radiat. Prot. Dosim..

[73] Qureshi A.A., Tariq S., Ud Din K., Manzoor S., Calligaris C., Waheed A. (2014). Evaluation of excessive lifetime cancer risk due to natural radioactivity in the rivers sediments of Northern Pakistan. J. Radiat. Res. Appl. Sci..

[74] Stevulova N., Junak J., Vaclavik V. (2018). Effect of Silica Fume as a Component of Alternative Binder on the Selected Technically Important Characteristics of Bio-Aggregate-Based Composites. Materials.

[75] Younis H., Ahmad F., Shehzadi R., Asghar I., Ahmad T., Ajaz M., Waqas M., Mehboob K., Qureshi A.A., Haj Ismail A.A.K. (2021). Study of Radioactivity in Bajaur Norite Exposed in the Himalayan Tectonic Zone of Northern Pakistan. Atmosphere.

